# Comparative pangenome analysis of capsulated *Haemophilus influenzae* serotype f highlights their high genomic stability

**DOI:** 10.1038/s41598-022-07185-5

**Published:** 2022-02-24

**Authors:** Aida Gonzalez-Diaz, Anna Carrera-Salinas, Miguel Pinto, Meritxell Cubero, Arie van der Ende, Jeroen D. Langereis, M. Ángeles Domínguez, Carmen Ardanuy, Paula Bajanca-Lavado, Sara Marti

**Affiliations:** 1 Microbiology Department, Hospital Universitari de Bellvitge, IDIBELL-UB, Feixa Llarga s/n, L’Hospitalet de Llobregat, 08907 Barcelona, Spain; 2 Research Network for Respiratory Diseases (CIBERES), ISCIII, Madrid, Spain; 3 Bioinformatics Unit, Department of Infectious Disease, National Institute of Health, Lisbon, Portugal; 4 Infection and Immunity Amsterdam, Department of Medical Microbiology and Infection Prevention, Amsterdam UMC, Amsterdam, The Netherlands; 5 Amsterdam UMC, Reference Laboratory for Bacterial Meningitis, Amsterdam, The Netherlands; 6 Section Pediatric Infectious Diseases, Laboratory of Medical Immunology, Radboud Institute for Molecular Life Sciences, Radboudumc, Nijmegen, The Netherlands; 7 Radboud Center for Infectious Diseases, Radboudumc, Nijmegen, The Netherlands; 8 Department of Pathology and Experimental Therapeutics, School of Medicine, University of Barcelona, Barcelona, Spain; 9 Research Network for Infectious Diseases (CIBERINFEC), ISCIII, Madrid, Spain; 10 Haemophilus Influenzae Reference Laboratory, Department of Infectious Disease, National Institute of Health, Lisbon, Portugal; 11 Department of Medicine, School of Medicine, University of Barcelona, Barcelona, Spain

**Keywords:** Bacteria, Microbial genetics, Genomics, Prokaryote

## Abstract

*Haemophilus influenzae* is an opportunistic pathogen adapted to the human respiratory tract. Non-typeable *H. influenzae* are highly heterogeneous, but few studies have analysed the genomic variability of capsulated strains. This study aims to examine the genetic diversity of 37 serotype f isolates from the Netherlands, Portugal, and Spain, and to compare all capsulated genomes available on public databases. Serotype f isolates belonged to CC124 and shared few single nucleotide polymorphisms (SNPs) (n = 10,999), but a high core genome (> 80%). Three main clades were identified by the presence of 75, 60 and 41 exclusive genes for each clade, respectively. Multi-locus sequence type analysis of all capsulated genomes revealed a reduced number of clonal complexes associated with each serotype. Pangenome analysis showed a large pool of genes (n = 6360), many of which were accessory genome (n = 5323). Phylogenetic analysis revealed that serotypes a, b, and f had greater diversity. The total number of SNPs in serotype f was significantly lower than in serotypes a, b, and e (p < 0.0001), indicating low variability within the serotype f clonal complexes. Capsulated *H. influenzae* are genetically homogeneous, with few lineages in each serotype. Serotype f has high genetic stability regardless of time and country of isolation.

## Introduction

*Haemophilus influenzae* is a Gram-negative coccobacillus of the *Pasteurellaceae* family that colonises the human nasopharynx and throat in more than 50% of children and 20–30% of adults, causing a wide range of infection from chronic respiratory disease to severe invasive diseases, such as bacteraemia and meningitis^1,2^. The capsule is an important virulence factor, though it is not present in all strains of *H. influenzae*. Six different capsular operons have been described that encode six unique polysaccharide capsules (a–f). Strains missing the genes for the capsular operon are known as non-typeable *H. influenzae* (NTHi)^3^.

Prior to the introduction of a successful *H. influenzae* serotype b conjugate vaccine, invasive disease caused by the serotype b was leading significant cause of morbidity and mortality, especially in cases of meningitis in children under 5 years of age, while NTHi was almost exclusively associated with upper and lower respiratory tract infection^4,5^. However, widespread vaccine implementation has produced an epidemiological shift, and currently, most invasive infections occur in elderly patients with underlying conditions and are mainly caused by NTHi, followed at some distance by non-type b serotypes^6^. Serotype f should be considered a leading non-type b serotype that causes adult invasive *H. influenzae* disease, such as bacteraemia, in Europe and the United States^7–10^.

*H. influenzae* shares its ecological niche with many commensal bacteria and potential pathogens^11^. The local environment influences the ability to exchange genetic material; species that interact with other bacteria generally experience higher recombination rates than microorganisms living in less diverse settings^12^. Population structure analysis has shown that capsulated strains are highly clonal and have a limited number of serotype-associated lineages. By contrast, NTHi appears to have discrete subpopulation structures, but genetic diversity that is ten times greater. This clonality could be related to the presence of capsules and the fact that capsulated strains are more commonly found in invasive disease^13,14^. However, few studies have been performed in large datasets of capsulated genomes.

In this study, we examine the genomic diversity of *H. influenzae* serotype f through whole genome sequencing (WGS), using a multicentre collection of colonising and invasive isolates from the Netherlands, Portugal, and Spain. We also compare the population genetics of all capsulated *H. influenzae* genomes available in the National Center for Biotechnology Information (NCBI) and European Nucleotide Archive (ENA) databases.

## Results

### Pangenome variability in *H. influenzae* serotype f among countries and by sample origin

Of the 37 sequenced *H. influenzae* serotype f isolates, 33 were collected from invasive sites, including blood samples (n = 30) and cerebrospinal, joint, and pleural fluid samples (n = 1, each). The remaining four isolates were obtained from oropharyngeal colonisation of healthy children^15^. In the MLST profile, all isolates belonged to CC124, with most being ST124 (n = 31) and the rest being single-locus variants of ST124, such as ST1739 (n = 2), ST106, ST2390, ST2366, and ST2391 (n = 1, each) (Supplementary Table S1). The coloniser ES-HICOv-HILNM (ST106) and invasive ES-HUB-11665 (ST2366) strains were phylogenetically related but did not show any epidemiological association because they were isolated from two different regions of Spain (Oviedo and Barcelona) separated by twelve years.

**Figure 1:**
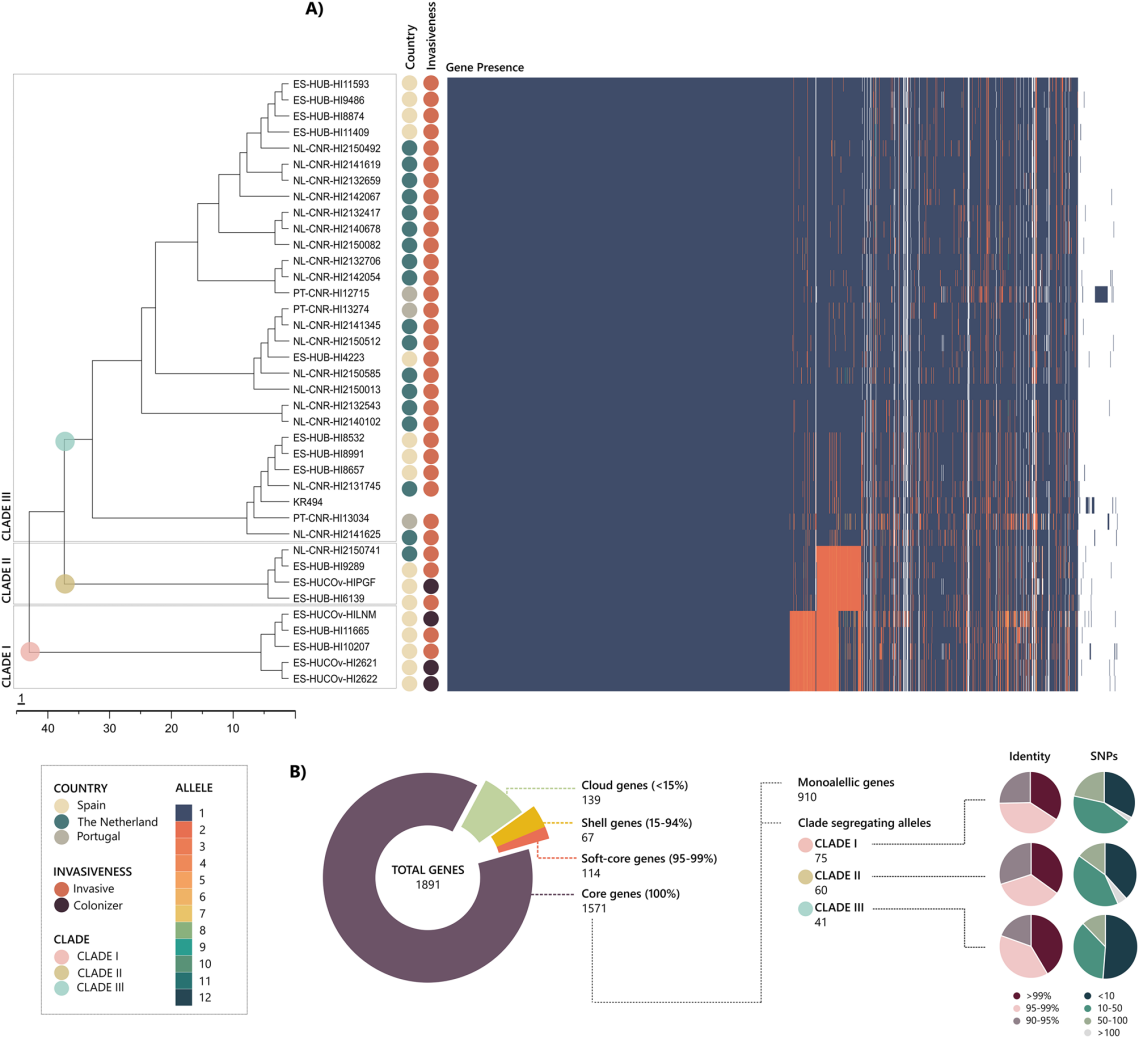
Pangenomic analysis of the 37 *H. influenzae* serotype f genomes. (**A**) Core-SNP phylogenetic tree, demographic data (country and invasiveness), genes detected, and the assigned allele. Clades I, II and III are indicated by coloured dots. The percentage of strains carrying each gene is presented graphically. (**B**) Distribution of genes detected in *H. influenzae* serotype f: core genes (100% of genomes), soft-core genes (95–99%), shell genes (15–94%), and cloud genes (< 15%). Core genes were classified as monoallelic (same allele in all the isolates) or clade segregating alleles (an allelic variant exclusive to one clade). The pie charts show the identity and number of SNPs for alleles of each clade in relation to the alleles of the same gene in other clades.

The proportion of the genome shared by the 37 isolates was very high (> 80%), and only 10,999 SNPs were found in the core genome, with an average of 1297 SNPs (SD = 1799) compared to the reference genome. After core genome phylogenetic analysis, three clades (I–III) were distinguished by the presence of clade-specific allelic variants represented (Fig. 1A). Sub-clades of clade III did not present exclusive alleles that allow it to be considered an independent clade. Clades I and II were less common (n = 5 and n = 4, respectively) and included the four coloniser isolates. Clade I contained two invasive and three colonising strains, all of them isolated in Spain. Clade II included the remaining coloniser isolates as well as three invasive isolates, two from Spain and one from the Netherlands. By contrast, clade III grouped most serotype f genomes (n = 28) and was exclusively associated with invasive clones. No phylogenetic association was found with the geographical origin of clade III clones.

Pangenome analysis detected 1891 genes in the gene pool, of which 1571 were present in all genomes (core genes), 114 genes in 95–99% (soft-core genes), 67 in 15–94% (shell genes), and 139 genes in < 15% (cloud genes) (Fig. 1B). Country, source or year of isolation, as well as clade, were not related to the presence or absence of any gene. Among the core genes, 910 were monoallelic (same allele in all isolates) and 499 had alleles distributed indifferently across the isolates and clades. In addition, 162 genes had clade-specific alleles that allowed segregation of the genomes into the different clades: clade I had 75 specific alleles, clade II had 60 alleles, and clade III had 41 alleles. When these alleles were compared to other alleles of the same gene, the identity and number of SNPs showed low variability (Fig. 1): clade I segregating alleles had a mean identity of 97.1% (standard deviation [SD] = 2.7) and an average of 32.0 SNPs (SD = 33.1); clade II segregating alleles had an identity of 96.8% (SD = 2.9) and 31.4 SNPs (SD = 34.4); and clade III segregating alleles had an identity of 97.6% (SD = 2.5) and 19.0 SNPs (SD = 24.6). However, it should be noted that clade III alleles showed fewer SNPs compared to clade I (p-value = 0.0291) and clade II (p-value = 0.0538) alleles. The genes associated with clade segregating alleles, their protein products, and their functions are shown in Supplementary Table S2.

### MLST and phylogenetic relationship of capsulated *H. influenzae*

A total of 800 genomes, comprising 763 of capsulated *H. influenzae* from the NCBI and ENA databases (Supplementary Table S3) and 37 of serotype f in this study, were included to determine the phylogenetic diversity of capsulated *H. influenzae*. The presence of capsular type in silico revealed 205 serotype a, 165 serotype b, 34 serotype c, 10 serotype d, 152 serotype e, and 234 serotype f genomes (197 from databases and 37 from this study).

The genomes of capsulated *H. influenzae* showed high homogeneity within each serotype. The phylogenetic analysis revealed three major branches, one containing the genomes of serotypes a, b, c, and d, one containing CC6 genomes of serotype b, and the other containing the genomes of serotypes e and f, as well as the CC464 genome of serotype b and genomes of serotype a associated to CC62 and CC372 (Supplementary Fig. S1). Each serotype had a distinct monophyletic lineage, except for serotypes a and b from the first branch, where CC4 and CC50, respectively, showed a different origin compared to other genomes of their serotypes.

Each serotype was classified into clades based on its phylogenetic origins and clonal complex distribution. Serotype a genomes were phylogenetically grouped into three different clades (Supplementary Fig. S2A). Those belonging to CC23 (n = 176) were grouped in a major clade that could be divided into two subclades, one mainly associated with ST23 and one that grouped single-, double- and triple-locus variants of ST23. CC1755 (n = 1), which only shared three loci of ST23, showed a close genetic relationship with this last subclade. The remaining genomes were grouped in two minor clades, one including CC62 (n = 18) and CC372 (n = 2) and another including CC4 (n = 8). Serotype b genomes mainly belonged to CC6 (n = 150) and formed one of the main groups based on phylogenetic analysis, together with an isolate from a new CC. Genomes from CC50 (n = 13) and CC464 (n = 1) constituted a minor phylogenetic group (Supplementary Fig. S2B). Serotype c and d genomes showed less genetic variability than other capsulated genomes, probably due to the low number of sequenced isolates. Serotype c genomes were linked to CC7, whereas serotype d genomes belonged to CC10 and were divided into two clades (Supplementary Fig. S3). Serotype e genomes were exclusively related to CC18, with ST18 being most abundant (n = 66) and were distributed in two different branches of the phylogenetic tree (Supplementary Fig. S4A). Serotype f genomes belonged to CC124 (n = 222) and CC16 (n = 12), which were distributed into major (clade I to III) and minor (clade IV) clusters, respectively. The 37 genomes from the Netherlands, Portugal, and Spain were distributed throughout the major cluster of the phylogenetic tree. The minor cluster showed a close phylogenetic relationship with two ST124 genomes (Supplementary Fig. S4B).

### Pangenomic analysis of capsulated *H. influenzae*

**Figure 2:**
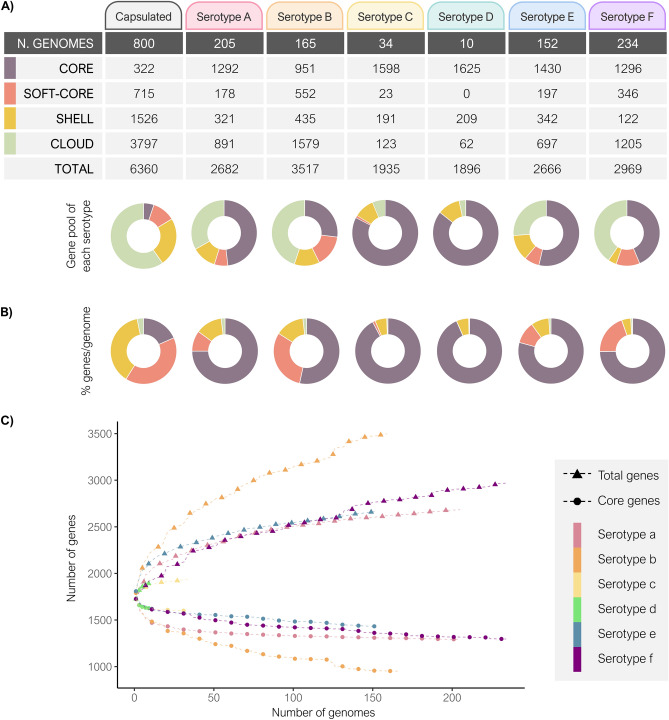
Pangenomic analysis of capsulated *H. influenzae*. (**A**) Gene pool of capsulated *H. influenzae* genomes included in this study. The number of core, soft-core, shell, cloud, and total genes of each serotype was determined using Roary, with a minimum identity percentage of 70% for BLASTp and the -cd parameter adjusted to 100. (**B**) Relative pangenome composition represented as a percentage of genes per genome of each serotype. Gene pool was defined as the set of all genes in a population. Donut charts indicate the distribution of core (100% of genomes), soft-core (95–99%), shell (15–94%), and cloud genes (< 15%). (**C**) Correlation between total and core genes in all capsulated *H. influenzae* genomes from this study and from the NCBI and ENA databases by serotype.

The analysis of 800 pangenomes revealed that the gene pool of capsulated *H. influenzae* included 6360 genes (Fig. 2A). The proportion of genes present in all genomes (core genes) was very low across the capsulated *H. influenzae* population, accounting for only 5.1% (n = 322), whereas 11.2% (n = 715) were identified in 95–99% of the genomes (soft-core genes). The accessory genome included 5323 genes, distributed in shell genes (n = 1526; 24%), present in 15–94% of genomes, and cloud genes (n = 3797; 59.7%), present in < 15% of genomes.

Differences in the gene pools were observed when serotypes were analysed separately (Fig. 2A). *H. influenzae* serotype b showed the largest gene pool (n = 3517) compared to the other serotypes, which oscillated between 1896 and 2969 genes. Moreover, serotype b had the lowest core genome proportion (42.7%) due to the large amount of cloud genes detected in the gene pool (44.9%). By contrast, serotypes c and d had a small gene pool that was probably due to the low proportions of sequenced isolates (1935 and 1896 genes, respectively) and high proportions of core genes (83.7% and 85.7%, respectively). Finally, serotypes a, e, and f showed a better balance between the number of core and accessory genes present in the gene pool (54.8–45.2%, 61.0–39.0%, and 55.3–44.7%, respectively).

Despite the high number of genes detected within the gene pool of capsulated *H. influenzae* (n = 6360), each genome had an average of 1752 genes (SD = 51), ranging from 1725 (SD = 41) in serotype a genomes to 1805 (SD = 49) in serotype e genomes. Figure 2B depicts the pangenome composition for each serotype in an average genome. On average, serotype b had more accessory genes per genome than the other serotypes (mean = 46.6%, SD = 1.4), followed by serotype f (mean = 25.3%, SD = 1.1), serotype a (mean = 25.1%, SD = 1.8), and serotype e (mean = 20.7%, SD = 2.1). Serotype a and b isolates had subpopulations with fewer shell genes and more cloud genes per genome than the rest of the isolates for these serotypes. In serotype a, that subpopulation was associated with CC62 and CC372 (clade II), with an average of 43 shell genes (SD = 3) and 195 cloud genes (SD = 14); this contrasted with the 245 shell genes (SD = 43) and 15 cloud genes (SD = 28) observed in the other CCs. In serotype b, genomes related to CC50 and CC464 (clade II) had 134 shell genes (SD = 22) and 98 cloud genes (SD = 49), while CC6 isolates (clade I) had 264 shell genes (SD = 29) and 27 cloud genes (SD = 46).

Differences in gene pool composition between serotypes, especially between serotype b and serotypes c and d, could be due to a disparity in the number of genomes. The association between the number of genomes and the gene pool composition is shown in Supplementary Fig. S5. As more genomes were included, the number of total genes increased due to the introduction of more accessory genes. Core genes, however, fell considerably with the inclusion of the first genomes to reach a stable plateau. Although all serotypes showed the same tendency, it was notable that the variation between core and total genes was higher for serotype b than for the other serotypes, indicating that accessory gene acquisition was greater in serotype b (Fig. 2C).

A detailed analysis of the genetic composition revealed the presence and absence of genes associated with each serotype (Supplementary Table S4). The presence of 32 genes were associated with serotype f genomes but not with other serotypes. On the other hand, two genes, which code for the 30S ribosomal subunit protein S15 and a predicted nucleotide binding protein, were mostly absent in serotype f but present in other serotypes (Supplementary Table S4).

### SNP typing of capsulated *H. influenzae*

The 800 capsulated *H. influenzae* genomes had 97,175 SNPs in the core genome, with an average of 26,626 SNPs (SD = 18,266) compared to the reference genome. Furthermore, serotype a (mean = 8499.5 SNPs, SD = 17,713.2), b (mean = 6048.2 SNPs, SD = 7472.1), and e (mean = 6849.4 SNPs, SD = 2895.3) genomes presented more SNPs than serotype f (mean = 2401.2, SD = 5206.9) genomes (p-value < 0.0001), whereas serotypes c (mean = 4037.1, SD = 4784.9) and d (mean = 6849.4 SNPs, SD = 2859.3) showed no significant differences despite having a greater number of SNPs than serotype f.

**Figure 3:**
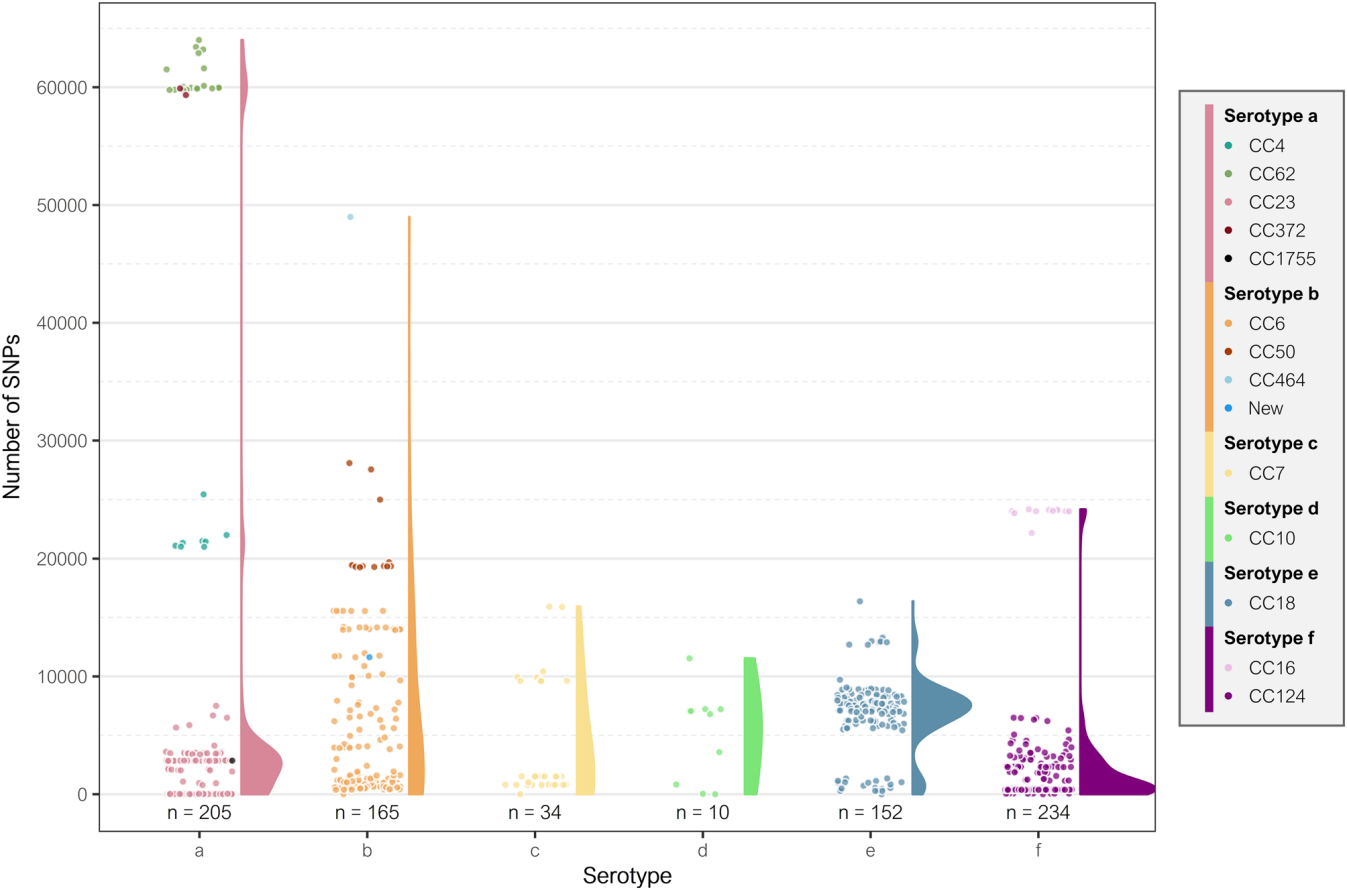
Core genome SNP typing of capsulated *H. influenzae* genomes. Each dot reflects the number of SNPs found in serotype a, b, c, d, e, and f genomes compared to the reference genomes NML-Hia-1 [CC23] (NZ_CP017811.1), 10810 [CC6] (NC_016809.1), M12125 [CC7] (SRR9847495), PTHi-10983 [CC10] (ERR2560729), M15895 [CC18] (NZ_CP031249.1), and KR494 [CC124] (NC_022356.1), respectively. Split violin plots show the distribution of the genomes based on the number of SNPs by each serotype.

However, the genetic variability within each serotype and the reference genome used in each case should be considered (Fig. 3). Genomes of each serotype could be classified in CCs by the number of SNPs observed. For serotype a genomes, NML-Hia-1 (NZ_CP017811.1) of CC23 was used as reference. The isolates from CC23 showed an average of 1988 SNPs (SD = 1598) compared to the reference genome. CC4 genomes presented more genetic differences, with an average of 21,858 SNPs (SD = 1492), while CC62 and CC372 were the less related to the CC23 reference genome, with averages of 60,864 SNPs (SD = 1498) and 59,621 SNPs (SD = 394), respectively. In serotype b, using 10,810 (NC_016809.1) from CC6 as the reference strain, most genomes were grouped in two clusters, one related to CC6 that had 4418 SNPs (SD = 5045) and one linked to CC50 that had 21,122 SNPs (SD = 3364). Finally, strain KR494 (NC_022356.1) from CC124 was used as the reference for serotype f, showing 1238 SNPs (SD = 1450) for CC124 compared with the greater genetic distance of 23,906 SNPs for CC16 (SD = 552).

## Discussion

The conjugate vaccine against serotype b has changed the global epidemiology of *H. influenzae*. NTHi is now the leading cause of invasive and non-invasive infection^16^, while serotype b is decreasing and serotype f, the most common capsulated serotype in invasive infections, is increasing among adult populations^17,18^. The severity of invasive serotype f infection is particularly notable because it can affect immunocompetent patients and results in more than one-third of patients being admitted to intensive care units^17^. For these reasons, we provide a pangenome analysis of serotype f isolates associated with colonisation and invasive disease and perform a comparison with other capsular serotypes of *H. influenzae*.

The analysis of colonising and invasive serotype f isolates revealed minimal genetic diversity, with all being related to ST124 or a few single-locus variants of ST124. Consistent with prior reports, this was irrespective of the country, year, or source of isolation^19,20^. Bruun et al.^21^, identified a long-term stable clone lineage in different countries for more than 50 years, and supporting their findings, our results suggest that this single clone (CC124) has persisted. Despite the low number of SNPs among the 37 isolates, which is consistent with the low number of SNPs found in serotype f genomes obtained from NCBI and ENA databases when compared to other serotypes, three distinct clades associated with CC124 could be identified. Clade III included most isolates and was exclusively associated with invasive strains and showed less variability than either clade I or II, which included the colonising strains. This suggests that the variability in serotype f genomes, despite being low, is mainly associated with colonising strains. According to a previous study, *Streptococcus pneumoniae*, which inhabits the same niche as *H. influenzae*, had high recombination rates that were linked to a longer colonisation status, favouring direct interactions with other bacteria of the same or different species^22^. For this reason, invasive clones may be more genetically homogeneous, while colonising strains may be more diverse due to higher genetic exchange with their environment.

The inclusion of all capsulated *H. influenzae* genomes available in the NCBI and ENA databases allowed comparison of the population structure for different serotypes and placed the genetic stability of serotype f in context. Moreover it provides an overview of the capsulated *H. influenzae* pangenome or supragenome, consisting of the entire set of genes available that is not contained by any particular isolate, but is available through a genetically diverse population. However, the lack of clinical data for the NCBI and ENA genomes precluded the identification of genetic differences between colonising and invasive strains. The pangenomic analysis of the capsulated genomes identified a pool of 6360 genes, with only 1037 being core or soft-core elements of genome. Pinto et al.^13^ reported a smaller gene pool, which could be explained by the lower number of genomes in their study. Moreover, regardless of the serotype, all isolates carried roughly the same number of genes per genome (mean = 1752, SD = 51), again consistent with previous findings^20^. The maintenance of the overall number of genes per genome and the high fraction of accessory genes detected in the gene pool suggest that capsulated *H. influenzae* strains have a balance between gene acquisition and loss that could serve as a reservoir for DNA exchange. Hogg et al*.*^23^, developed a supragenome model that predicted a species-level pangenome of 5000 or more genes, as well as a core-genome of about 1400 genes. However, models are estimations that require the analysis of many strains to be confirmed. Despite the fact that they used NTHi strains, the pangenomic richness observed in the capsulated strains of our study are consistent with their model. This diversity ensures the survival of the entire population in different environments, rather than the survival of an individual organism^24^.

Despite the pangenomic differences in the overall capsulated population, each serotype included a phylogenetically highly clonal population related to a few STs. Similarly, previous studies demonstrated low genetic diversity within each serotype by multi-locus enzyme electrophoresis, pulsed-field gel electrophoresis, MLST, and WGS^25–28^, suggesting that each serotype emerged once within the population^3^. However, despite the observed homogeneity of capsulated isolates, serotypes a and b displayed more variability in the MLST classification, with some STs differentiated by all seven loci, as Potts et al*.*^28^ previously observed. According to the observed heterogeneity in serotypes a and b, the overall phylogenetic analysis revealed that these serotypes had distinct lineages compared to the monophyletic origins observed in the other serotypes. Serotype b also exhibited greater pangenomic diversity than the other serotypes, having the largest gene pool and the highest proportion of accessory genome. This diversity could be attributed to several advantages of serotype b isolates over other serotypes, including the production of haemocin, a bacteriocin active against non-type b serotypes^26^, and the ability to evade the complement system^29^. These benefits would favour respiratory tract colonisation and genetic exchange by serotype b strains, promoting genetic diversity. However, the introduction of the conjugate vaccine likely resulted in less colonisation by, and less invasive disease due to, these organisms^18^.

Serotypes a, e, and f showed more diversity due to the accessory genome than serotypes c and d, but less than serotype b. This genetic diversity might promote bacterial survival in different ecological niches^24^, potentially explaining the successful emergence of invasive disease caused by these serotypes since conjugate vaccine introduction^19,30^. Nevertheless, infections caused by serotypes a, e, and f are rare, suggesting that there is no strain replacement^16^, probably due to their limitations in colonising the oropharynx. However, the genetic stability displayed by some clones, such as CC124 (serotype f) due to a low number of SNPs or CC23 (serotype a) due to a lower number of cloud genes, may be advantageous and may explain why these clones are more abundant and successful than other clones of these same serotypes^20,30,31^. Nevertheless, the addition of genomes from less abundant clonal complexes or changes in the used reference genomes could modify the overall number of SNPs and the genetic variability observed in each serotype, although the differences between clonal complexes of the same serotype would be conserved. Serotypes c and d, presented low variability and a more abundant core genome than the other serotypes. However, the pangenome composition of these serotypes is still unclear and further studies are needed to elucidate this question, because they are rarely isolated and the number of sequenced strains is low.

Distinguishing serotype f isolates from other serotypes, apart from capsular genes, could be useful in developing therapeutic strategies against this emerging serotype. This study provides a first approximation of the genetic determinants associated with each of the serotypes (Supplementary Table S4). However, the methodology used has certain limitations, as it is possible that truncated, duplicated, or those genes broken in different contigs would not be included. Thus, further studies are required to improve the identification of these genetic determinants.

In contrast to capsulated isolates, NTHi shows high genetic heterogeneity^32^. *H. influenzae* is a transformable bacterium for which homologous recombination enhances genetic diversity, affecting the commensal and the virulent behaviour of the microorganism^33^. Some studies have demonstrated that the level of recombination in NTHi was greater than in typeable isolates and that the polysaccharide capsule reduces the rate of gene transfer^27,34^, probably due to its role as a physical barrier. In addition, colonisation is more commonly associated with NTHi than with capsulated isolates^35^. This would explain why NTHi clones have a high genetic heterogeneity while capsulated clones, which less frequently colonise the respiratory tract, have lower genetic heterogeneity.

## Conclusion

Capsulated *H. influenzae* isolates present high genomic homogeneity with few lineages per serotype. The genetic stability of invasive serotype f genomes, regardless of time and country of isolation, highlights the importance of genetic homogeneity in the clonal expansion of this serotype.

## Materials and methods

### Study design and bacterial strains

Retrospective laboratory-based multicentre study on the genomic diversity of invasive *H. influenzae* serotype f isolates. Heterogeneity across countries was examined using serotype f isolates collected between 2009 and 2014 from National Reference Centres in the Netherlands (n = 18) and Portugal (n = 3) and from Bellvitge University Hospital in Spain (n = 12) (Supplementary Table S1). All were isolated from sterile sites, including blood, cerebrospinal fluid, joint fluid, and pleural fluid, and were serotyped according to the Centres for Disease Control and Prevention (CDC) guidelines (http://www.cdc.gov/meningitis/lab-manual/chpt10-pcr.html). Colonising *H. influenzae* serotype f strains (n = 4) from Spanish children attending a day care centre^15^ were also included to establish the genetic differences between invasive and colonising isolates.

### Whole genome sequencing of *H. influenzae* serotype f isolates

WGS was performed for 37 *H. influenzae* serotype f isolates. Strains were grown on chocolate agar plates (bioMérieux, Marcy l’Etoile, France) and incubated at 37 °C in 5% CO_2_. Genomic DNA was extracted using the QIAamp DNA Mini Kit (Qiagen, Hilden, Germany) and quantified with the QuantiFluor^®^ dsDNA System (Promega, Wisconsin, USA). Libraries were prepared using Nextera XT and paired-end sequenced (2 × 150 base pairs) on a MiSeq Platform (Illumina Inc., San Diego, CA, USA), following the manufacturer’s instructions.

Read quality assessment and genome assembly was done using the INNUca v4.2 pipeline (https://github.com/B-UMMI/INNUca). Briefly, a quality control of the reads was performed using FastQC (http://www.bioinformatics.babraham.ac.uk/projects/fastqc), followed by a read cleaning and trimming with Trimmomatic^36^. The genome was assembled using SPAdes^37^ and was polished by Pilon^38^. In silico serotyping was conducted using hicap v1.0.3 (https://github.com/scwatts/hicap)^3^. The multi-locus sequence type (MLST) was determined in silico using the MLST v2.4 software (https://github.com/tseemann/mlst), and the new allele and sequence type (ST) numbers were registered in PubMLST (https://pubmlst.org). Genomes were classified into clonal complexes (CC) that included STs sharing at least five of the seven MLST alleles. The sequence reads were deposited at the ENA under the project accession number PRJEB45630 (Supplementary Table S1).

### Core and accessory genome analysis of *H. influenzae* serotype f isolates

Core single nucleotide polymorphisms (SNPs) were extracted with Snippy’s core module (snippy-core) for phylogenetic analysis, using the default parameters and the *H. influenzae* KR494 (NC_022356) genome as a reference. Subsequently, the whole genome alignment was subjected to the prediction and removal of recombinant regions using the Gubbins v2.3.1 software^39^. A novel core-SNP phylogenetic tree was built in RAxML-NG^40^ based only on shared positions after recombination removal.

To characterise the genetic composition of the identified clades, the assembled genomes were annotated using Prokka v1.13.7^41^ and pangenome analysis was done using Roary^42^ with a minimum identity percentage of 70% for BLASTp, as previously described for this species^13^, and the -cd parameter adjusted to 100. Allelic profiles were determined using roProfile (https://github.com/cimendes/roProfile) where alleles with size variation > 20% were discarded by default.

### Analysis of population genetics for capsulated *H. influenzae* genomes

To better understand the phylogenetic diversity in capsulated *H. influenzae*, all capsulated genomes available in the NCBI and ENA databases were downloaded and selected (see Supplementary Fig. S6). Pre-selection of ENA genomes was performed by mapping reads against the *bexA* gene using Bowtie2^43^. A total of 763 genomes were identified as capsulated *H. influenzae* after in silico serotyping and MLST classification.

SNPs were studied with Snippy, using default parameters, and were visualised using the ggplot2 R package^44^. Phylogenetic analysis was performed using Snippy’s core module and Gubbins v2.3.1 software, as described above. Pangenome analysis was done with Roary, and a gene pool was defined as the set of all genes detected in a population. A statistical analysis was performed based on the Roary results to determine the presence and absence of genes associated with serotype f. Thus, Scoary (https://github.com/AdmiralenOla/Scoary) was used for the analysis, and genes with specificity and sensitivity > 97.5% and < 2.5% were chosen to select the presence and absence of genes, respectively.

The genomes of strains NML-Hia-1 (NZ_CP017811.1, CC23)^45^, 10810 (NC_016809.1, CC6)^20^, M12125 (SRR9847495, CC7), PTHi-10983 (ERR2560729, CC10), M15895 (NZ_CP031249.1, CC18), and KR494 (NC_022356.1, CC124)^46^, which belonged to the clinically most prevalent clonal complexes of each serotype, were used as references for serotypes a to f, respectively. In the overall analysis of capsulated *H. influenzae*, the genome of KR494 (NC_022356.1) was used as a reference.

### Statistical analysis

Statistical analyses were performed in GraphPad Prism 5, using unpaired *t* test or one-way ANOVA (Newman–Keuls test), as appropriate. P-values < 0.05 were considered statistically significant.

### Ethical approval

This study was in accordance with the Declaration of Helsinki from the World Medical Association. Written informed consent was not required as this was a retrospective and observational study with isolates obtained as part of routine microbiological tests, which was approved by the Clinical Research Ethics Committee of Bellvitge University Hospital (PR334/21). Patient confidentiality was always protected, and all personal data were anonymised following the current legal normative in Spain (LOPD 15/1999 and RD 1720/2007). Moreover, this project followed Law 14/2007 on Biomedical Research for the management of biological samples in clinical research.

### Repositories

Sequence reads were deposited in the European Nucleotide Archive (ENA) under the project accession number PRJEB45630.

## Supplementary Information

Supplementary Figures.

Supplementary Table S1.

Supplementary Table S2.

Supplementary Table S3.

Supplementary Table S4.
