# Supplementary material for: Comparative pangenome analysis of capsulated Haemophilus influenzae serotype f highlights their high genomic stability
Source: Sci Rep. 2022 Feb 24;12:3189. doi: 10.1038/s41598-022-07185-5 (PMC8873416; doi:10.1038/s41598-022-07185-5)
Supplement: Supplementary file 1 — Supplementary Figures. [file 41598_2022_7185_MOESM1_ESM.pdf]

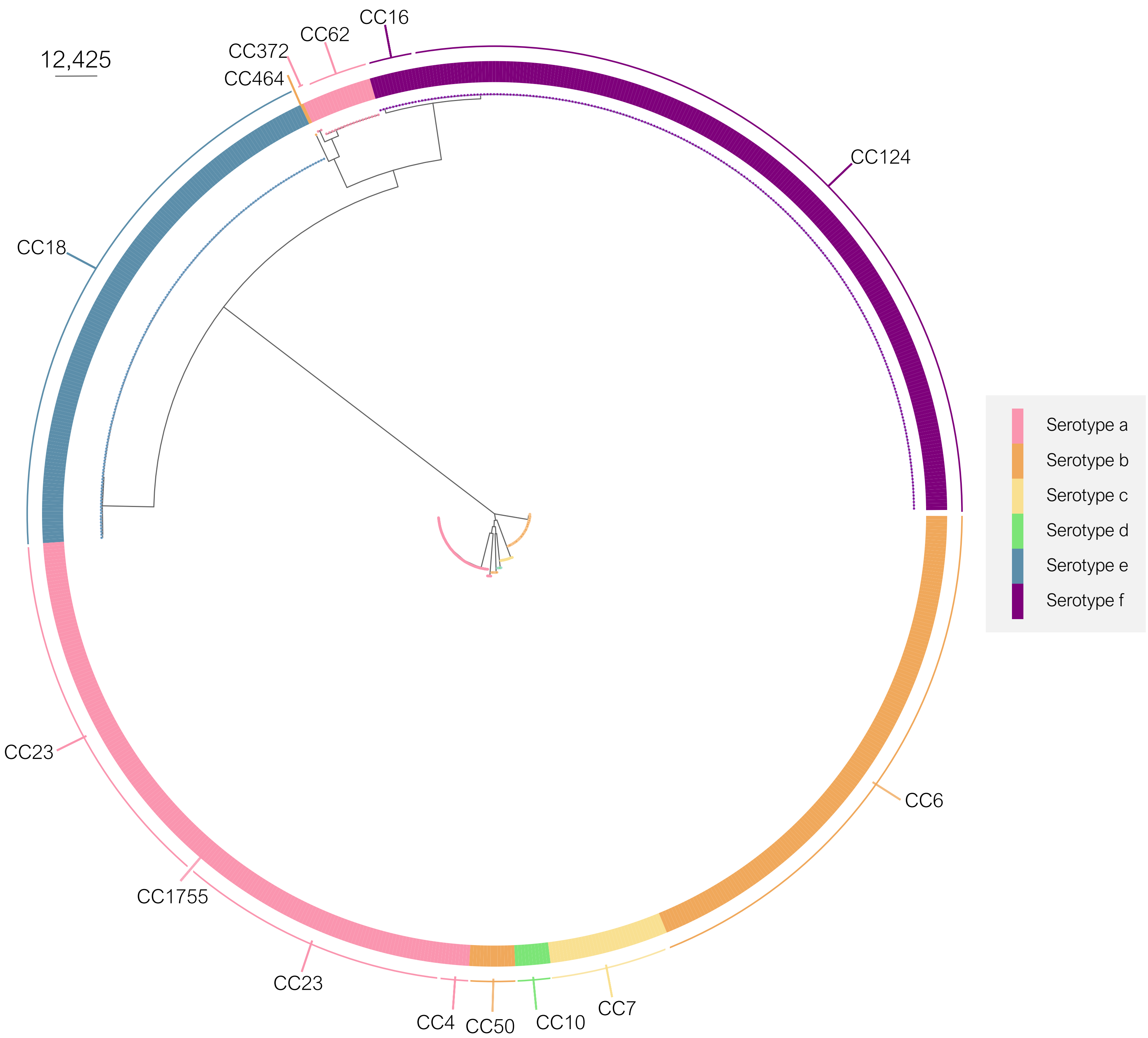

Supplementary Figure S1. Phylogenetic core-SNP tree of capsulated *H. influenzae* isolates.

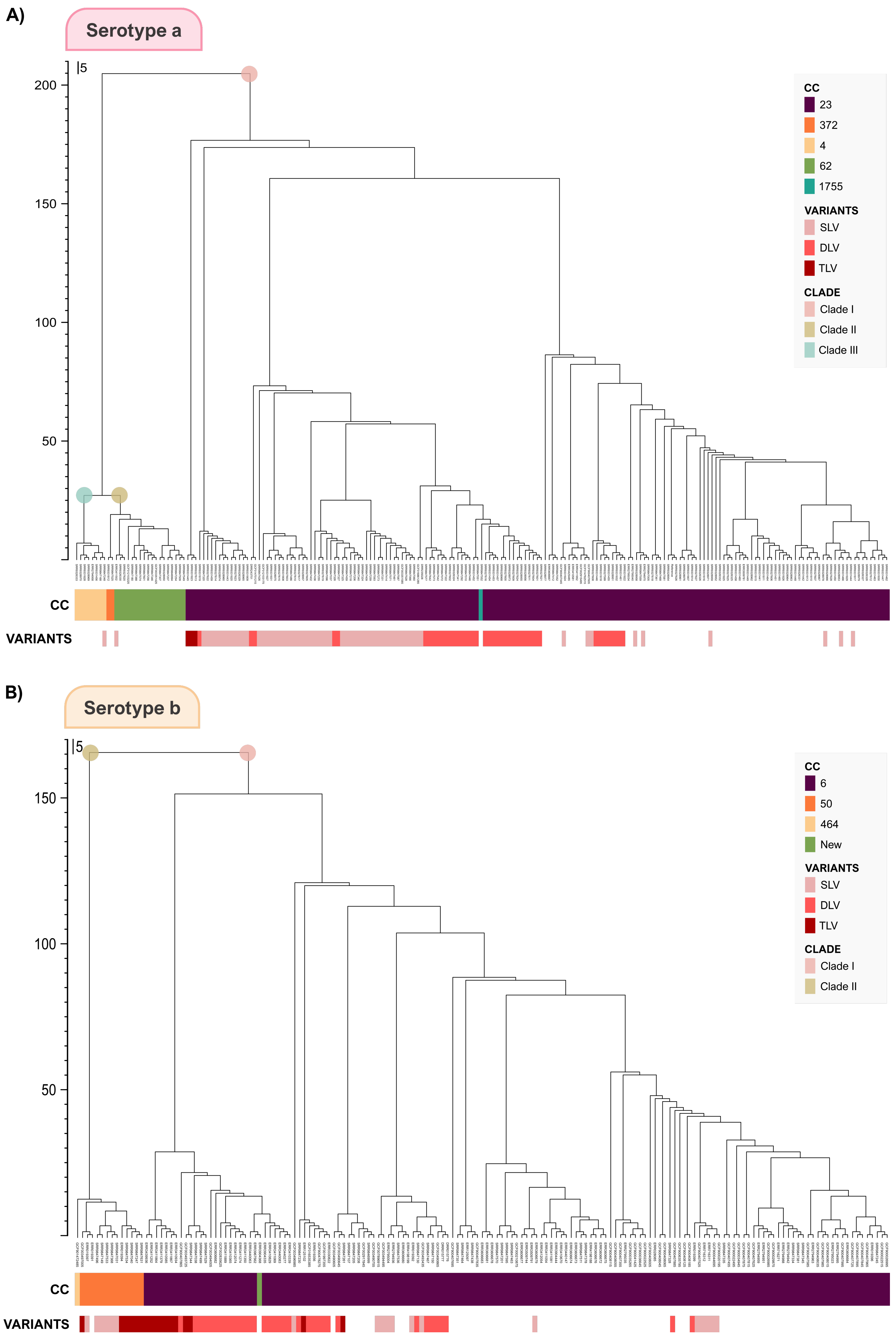
A)
Serotype a
5
200
150
100
50
CC
23
372
4
62
1755
VARIANTS
SLV
DLV
TLV
CLADE
Clade I
Clade II
Clade III
CC
VARIANTS
B)
Serotype b
5
150
100
50
CC
6
50
464
New
VARIANTS
SLV
DLV
TLV
CLADE
Clade I
Clade II
CC
VARIANTS

Supplementary Figure S2. Phylogenetic core-SNP tree of *H. influenzae* serotype a (A) and b (B). Genomes were classified into CCs, defined as sequence types sharing at least five of seven MLST alleles. STs differing at one, two, or three of the seven loci were defined as SLVs, DLVs and TLVs, respectively. Circles in the phylogenetic trees group genomes into clades. Abbreviations: CC, clonal complexes; MLST, multi-locus sequence type; SNP, single nucleotide polymorphism; ST, sequence type; SLV single-locus variant; DLV, double-locus variant; TLV, triple-locus variant.

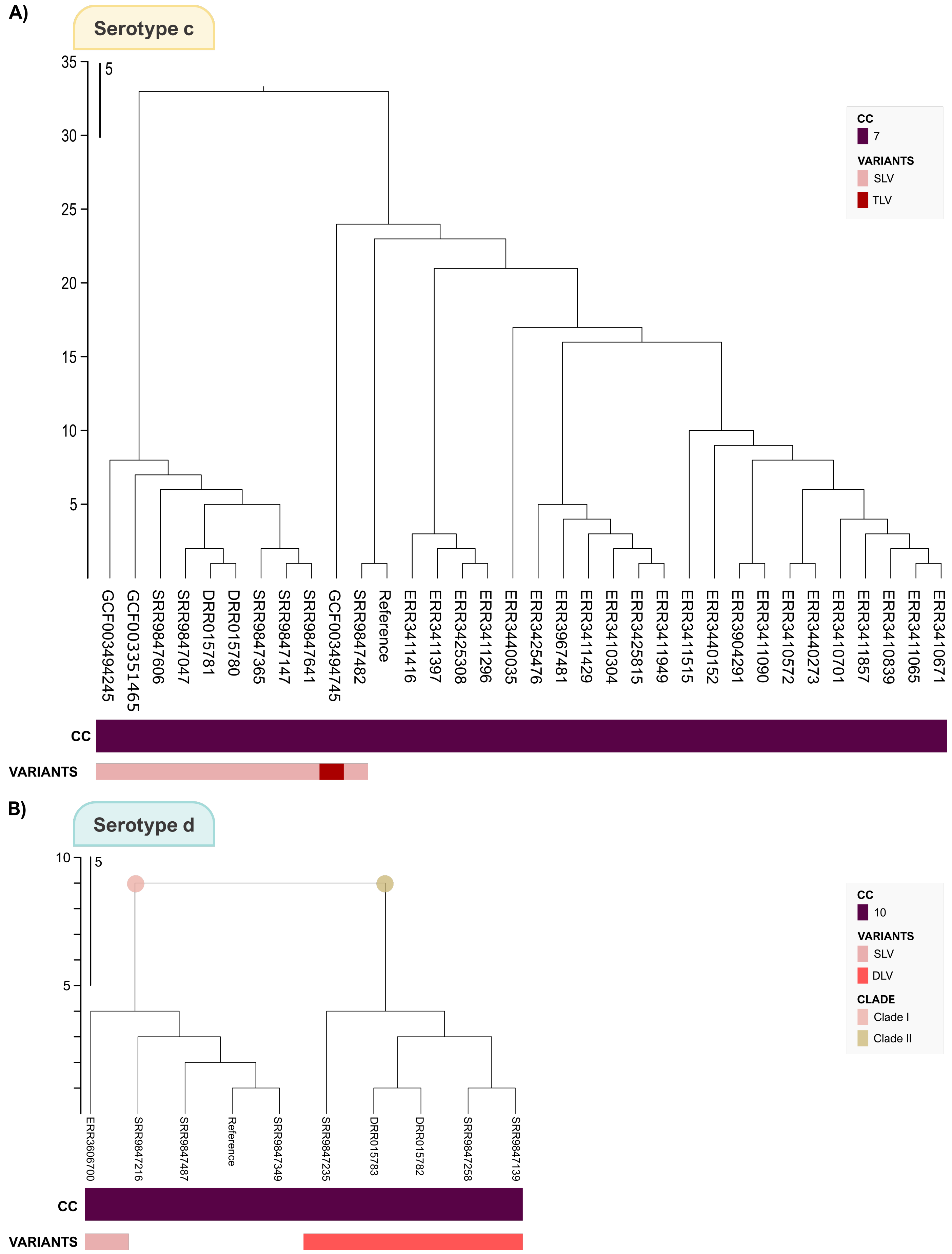

Supplementary Figure S3. Phylogenetic core-SNP tree of *H. influenzae* serotype c (A) and d (B). Genomes were classified into CCs, defined as sequence types sharing at least five of seven MLST alleles. STs differing at one, two, or three of the seven loci were defined as SLVs, DLVs, and TLVs, respectively. Circles in the phylogenetic trees group genomes into clades. Abbreviations: CC, clonal complexes; MLST, multi-locus sequence type; SNP, single nucleotide polymorphism; ST, sequence type; SLV single-locus variant; DLV, double-locus variant; TLV, triple-locus variant.

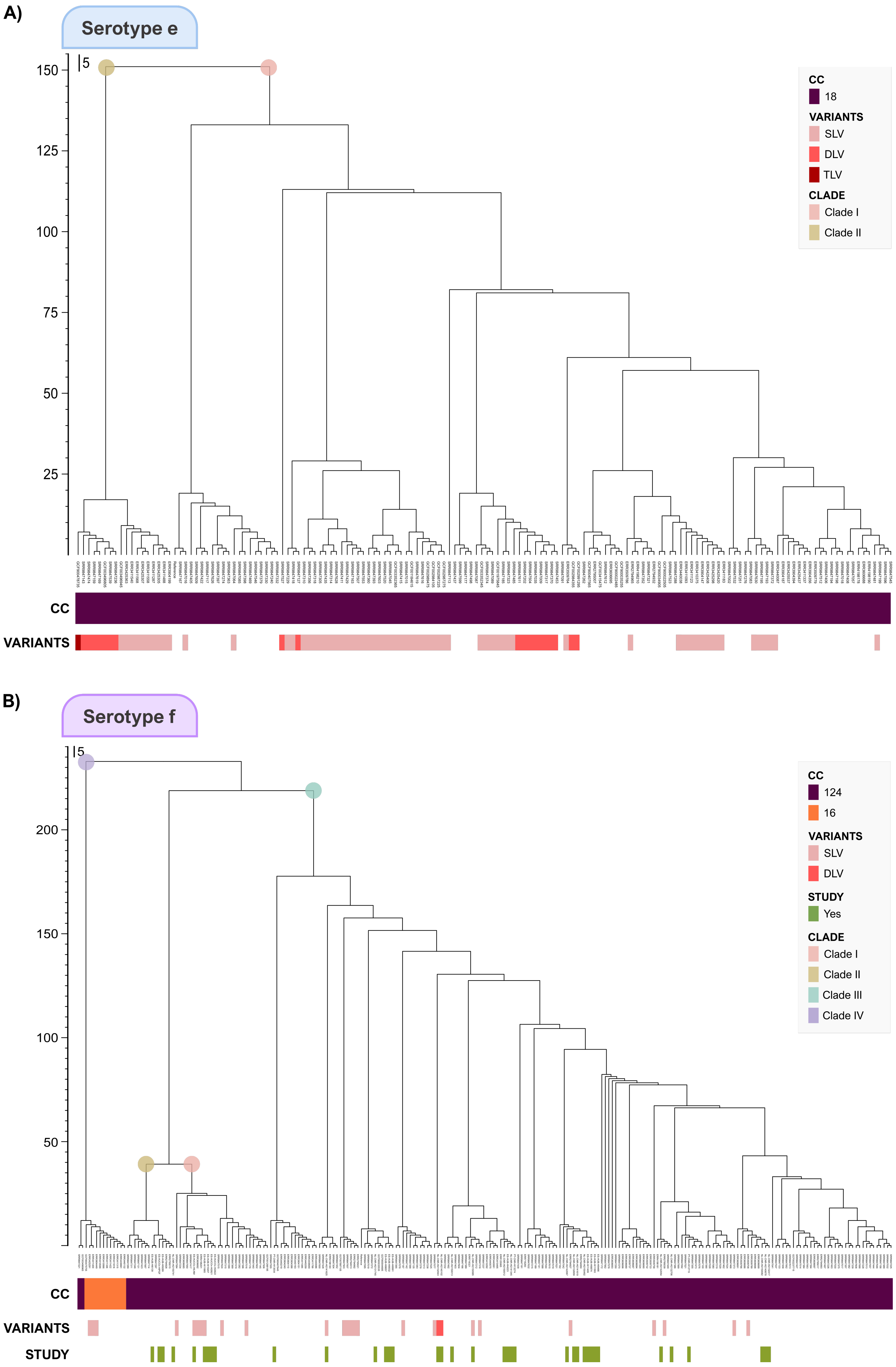
A)
Serotype e
5
150
125
100
75
50
25
CC
18
VARIANTS
SLV
DLV
TLV
CLADE
Clade I
Clade II
CC
VARIANTS
B)
Serotype f
5
200
150
100
50
CC
124
16
VARIANTS
SLV
DLV
STUDY
Yes
CLADE
Clade I
Clade II
Clade III
Clade IV
CC
VARIANTS
STUDY

Supplementary Figure S4. Phylogenetic core-SNP tree of *H. influenzae* serotype e (A) and f (B). Genomes were classified into CCs, defined as sequence types sharing at least five of seven MLST alleles. STs differing at one, two, or three of the seven loci were defined as SLVs, DLVs, and TLVs, respectively. Circles in the phylogenetic trees group genomes into clades. Abbreviations: CC, clonal complexes; MLST, multi-locus sequence type; SNP, single nucleotide polymorphism; ST, sequence type; SLV single-locus variant; DLV, double-locus variant; TLV, triple-locus variant.

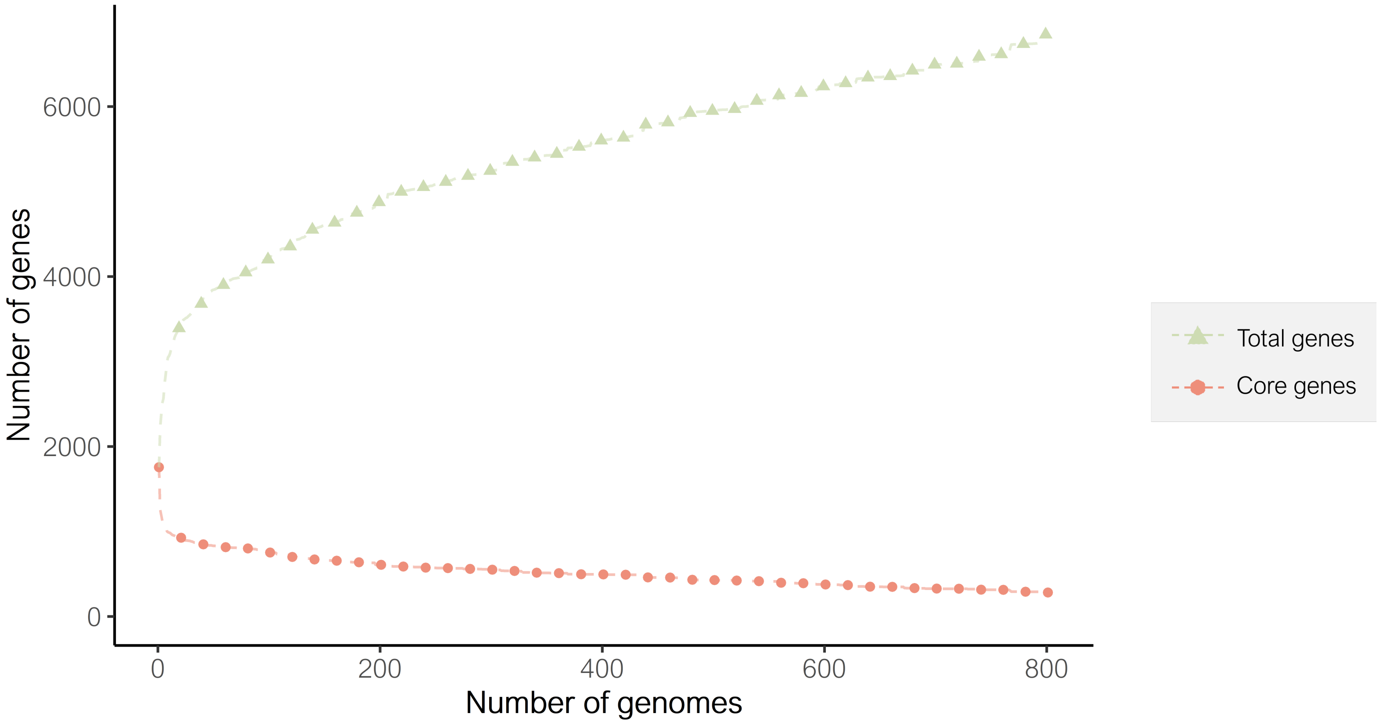

Supplementary Figure S5. Correlation between total and core genes in all capsulated *H. influenzae* genomes from this study and from the NCBI and ENA databases.

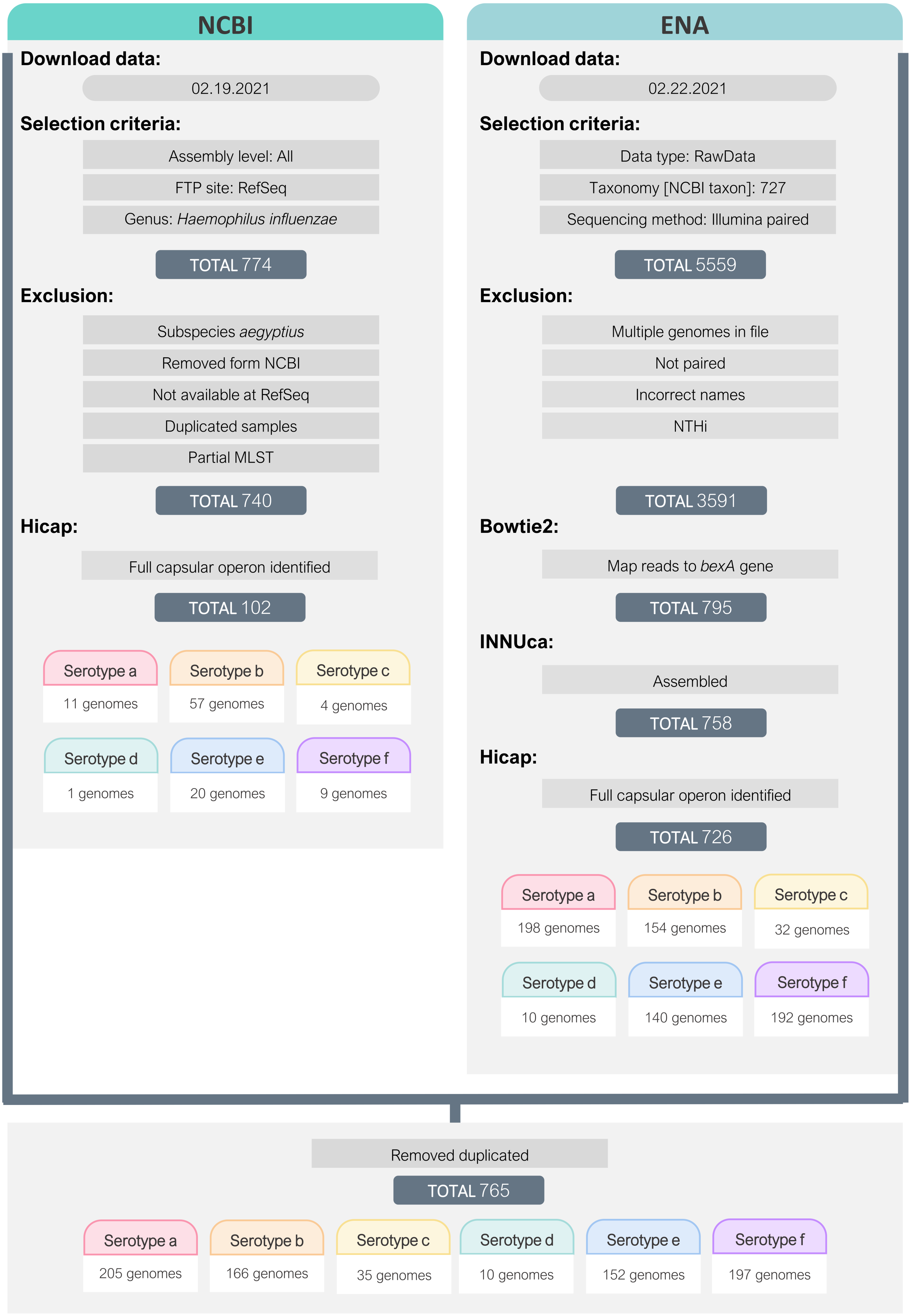

Supplementary Figure S6. Flowchart of the study pipeline to select the genomes of capsulated *H. influenzae* available on the NCBI and ENA databases. Abbreviations: ENA, European Nucleotide Archive; MLST, multi-locus sequence type; NCBI, National Center for Biotechnology Information.
